# Substrate temperature effects on structure and thermoelectric transport in DC-sputtered Bi_2_Te_3_ thin films

**DOI:** 10.1038/s41598-026-42427-w

**Published:** 2026-03-10

**Authors:** Mohammad Mahdi Shahidi, Yasaman Saberi Kakhki, Mohammad Amir Bazrafshan, Razieh Morad, Mahmood Akbari, Malik Maaza

**Affiliations:** 1 UNESCO-UNISA-ITL/NRF Africa Chair in Nanoscience and Nanotechnology, College of Graduate Studies, University of South Africa (UNISA), Muckleneuk Ridge, P.O. Box 392, Pretoria, South Africa; 2 School of Mechanical and Manufacturing Engineering, University of New South Wales, Sydney, NSW 2052 Australia

**Keywords:** Bi_2_Te_3_ thin films, DC magnetron sputtering, Substrate temperature, Thermoelectric properties, Power factor, Seebeck coefficient, Energy science and technology, Materials science, Nanoscience and technology, Physics

## Abstract

The thermoelectric performance of Bi₂Te₃ thin films is highly sensitive to deposition temperature because substrate heating simultaneously controls microstructure, stoichiometry, and charge transport. Here, ~ 450 nm Bi₂Te₃ films were deposited on glass by single-target DC magnetron sputtering while varying the substrate temperature from room temperature to 300 °C (RT, 100 °C, 200 °C, 300 °C). FESEM reveals progressive grain coarsening and improved grain connectivity with increasing temperature, while cross-sectional imaging confirms a comparable film thickness across all samples. EDS shows a monotonic Te-loss trend at elevated substrate temperatures, indicating increasing deviation from stoichiometric Bi₂Te₃. XRD confirms crystalline Bi₂Te₃ formation for all conditions and shows systematic peak shifts/lattice-spacing trends with temperature; no distinct elemental Bi or Te peaks are detected within measurement limits. Hall and transport measurements demonstrate that electrical conductivity increases with substrate temperature, whereas the Seebeck response decreases in magnitude, reflecting the expected conductivity–thermopower trade-off as composition and carrier transport evolve. The power factor ($PF = S^{2} \sigma$) reaches a maximum of ~ 4 µW cm⁻¹ K⁻² for films deposited at 200 °C, identifying an intermediate-temperature window that optimizes the balance between conductivity and thermopower for this DC-sputtering route. UV–Vis reflectance analysis indicates a substrate-temperature-dependent increase in the apparent optical bandgap extracted from Kubelka–Munk/Tauc-type treatment in the 300–1100 nm window, which is reported as an apparent optical metric rather than the intrinsic Bi₂Te₃ bandgap. Overall, these results establish ~ 200 °C as the most favorable substrate temperature in this study for achieving high PF Bi₂Te₃ thin films on glass and provide practical processing guidance for thermoelectric thin-film optimization.

## Introduction

Thermoelectric (TE) materials convert temperature gradient into electrical power via the Seebeck effect, enabling compact solid-state routes for waste-heat recovery and on-chip cooling applications^1–3^. Among near-room-temperature TE systems, Bismuth telluride (Bi₂Te₃)-based compounds remain a practical benchmark because they offer a favorable combination of thermopower, electrical conductivity, and relatively low thermal conductivity^4–7^. For thin films, additional microstructural levers such as crystallographic texture, grain size, interface density, and defect chemistry can markedly reshape carrier and phonon transport, allowing the power factor ($PF = S^{2} \sigma$) and, when $\kappa$is available, the figure of merit $ZT = S^{2} \sigma T/\kappa$ to be tuned through processing^8–13^.

Magnetron sputtering is widely adopted for Bi₂Te₃ thin films because it is scalable, thickness-controllable, and compatible with device integration on diverse substrates^14–16^. Within sputtering, the substrate temperature (${T}_{sub}$) is a first-order control knob: it sets adatom mobility and therefore governs crystallinity, texture development, grain coarsening, and the balance of native point defect (e.g., Te vacancies and Bi/Te antisites), while simultaneously competing with the volatility of Te^17,18^. As a result, moderate heating often improves ordering and carrier mobility (raising $\sigma$), whereas excessive heating can promote Te loss and stoichiometry drift, which typically penalizes $S$ and degrade the conductivity-thermopower balance that determines $PF$^19,20^.

Consistent “intermediate-temperature optimum” behavior has been reported across several sputtered Bi–Te and Bi₂Te₃ studies, but the optimal window depends strongly on the deposition route (DC vs. RF, single-target vs. co-sputtering), substrate choice, thickness, and whether post-treatments are used to recover texture or compensate Te loss. For example, co-sputtered Bi–Te films show an optimum around ~ 225–250 °C, while higher temperatures can trigger parasitic phases and collapse thermopower^21^. For DC-sputtered Bi₂Te₃ on inorganic substrates, elevated in-situ temperatures can increase σ yet al.so promote Te deficiency, motivating post-annealing strategies to restore texture and PF^22^. On flexible polyimide, pre-heating strengthens (00ℓ) texture and can enhance PF, but the highest thermal budgets again trend toward lower Te content^23^. Related thermal schedules (controlled anneal ramps, in-situ anneals, or Joule treatments) further underline how sensitive Bi₂Te₃ transport is to the thermal budget and stoichiometry management during and after growth^24–27^.

To place the present work in context and motivate its scope, Table 1 summarizes representative sputtered Bi₂Te₃ reports that explicitly vary ${T}_{sub}$ (or closely related thermal profiles such as pre-heating, dynamic heating, or post-annealing). Across these studies, moving from room temperature toward ~ 200–250 °C generally strengthens texture and improves σ while maintaining useful S; pushing toward ~ 300 °C can further coarsen grains, but increasingly risks Te loss and Seebeck suppression unless composition is actively stabilized.

Here, we deposit ~ 450 nm Bi₂Te₃ thin films on glass by single-target DC sputtering while sweeping ${T}_{sub}$ from RT to 300 °C, without post-annealing. Using XRD, FESEM/EDS, and temperature-dependent transport measurements, we build a temperature-resolved growth–structure–composition–transport map and quantify how ${T}_{sub}$ shifts the Seebeck–conductivity trade-off. We identify ${T}_{sub}$ ≈ 200 °C as the most favorable window for this processing route: σ increases with ${T}_{sub}$, the Seebeck coefficient is penalized at the highest ${T}_{sub}$ consistent with a Te-loss trend, and the maximum PF of ~ 4 µW cm⁻¹ K⁻² is achieved at ~ 200 °C. These results provide practical, substrate-specific processing guidance for DC-sputtered Bi₂Te₃ on glass and benchmark the achievable PF for a simple single-target route against the broader sputtering literature summarized in Table 1.

**Fig. 1:**
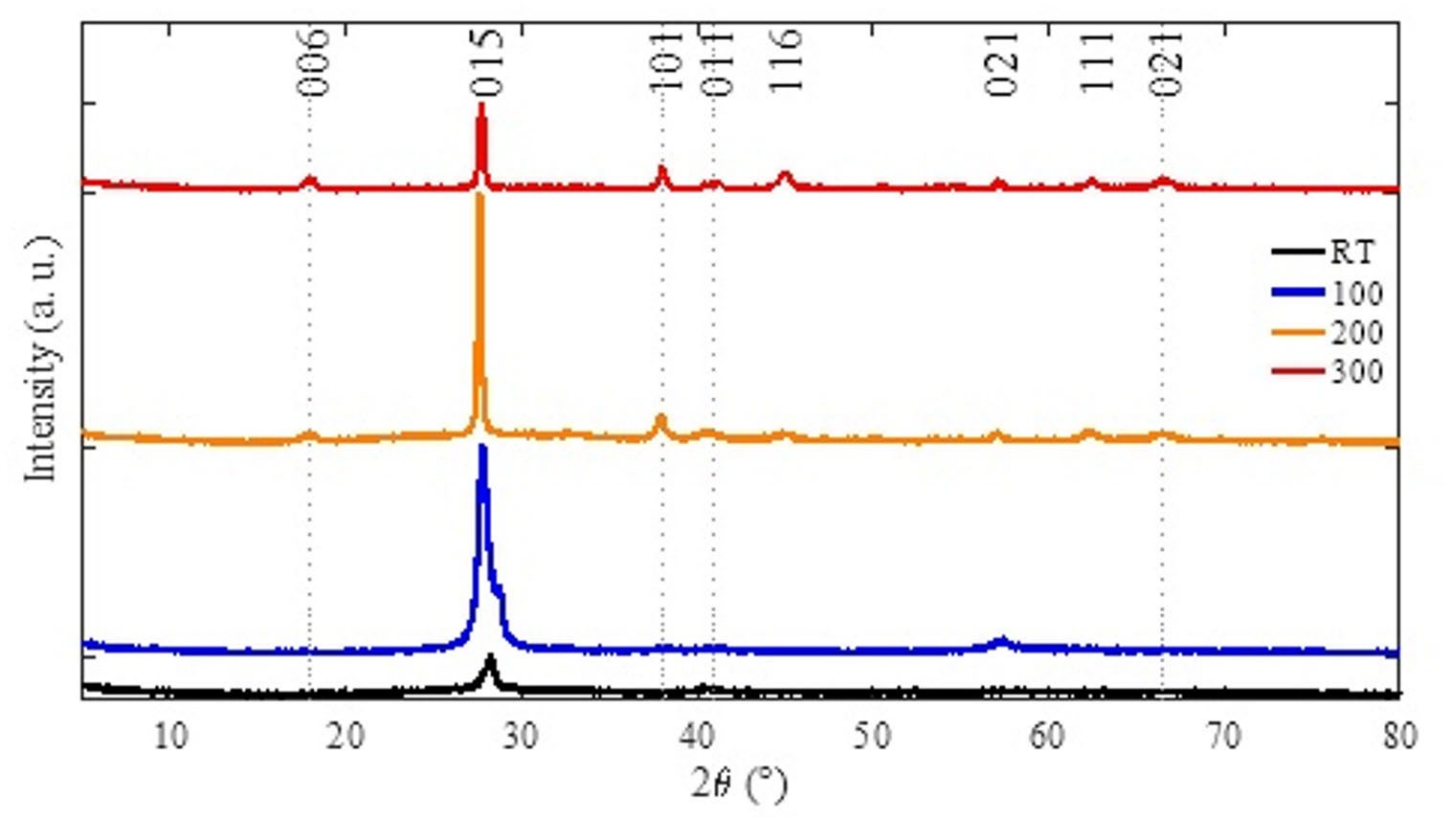
The XRD spectra of the samples. Vertical dotted lines indicate standard Bi_2_Te_3_ reflections according to 00-015-0863;00-042-1268.

**Table 1:** Effect of substrate temperature on Bi₂Te₃ films by DC/related sputtering (≈ RT values unless noted).

Study (year)	Method & thermal schedule	Substrate & thickness	Structure/stoichiometry vs. ${T}_{sub}$	TE metrics (σ, S, PF)	Key takeaway
This work	DC, Bi₂Te₃ (single target); ${T}_{sub}$ = RT/100/200/300°C; no post-anneal	Glass; ~450 nm	200 °C: faceted grains, improved texture; 300 °C: porosity and Te-loss trend	$\sigma$ ↑ with ${T}_{sub}$; $S$ ↓ at 300 °C; $PF$ ≈ 4 µW cm⁻¹ K⁻² @ 200 °C	~ 200 °C is $PF$-optimal; highest ${T}_{sub}$ penalizes S consistent with Te deficiency
Kim (2006)^21^	RF co-sputter (Bi + Te); ${T}_{sub}$ ≈ 140–430 °C (opt. ~225–250 °C)	Si/SiO₂; ~1 μm	Higher ${T}_{sub}$ reduces antisites; >~290 °C BiTe forms and $S$ collapses	$\sigma$ ~800–1000 S cm⁻¹ (opt.); $S$up to ~ − 120 µV K⁻¹; $PF$ ~20–25 µW cm⁻¹ K⁻²	Classic intermediate-${T}_{sub}$ optimum; high ${T}_{sub}$ risks parasitic phases
Kurokawa (2020)^22^	DC, Bi₂Te₃; heated deposition ~ 300 °C vs. RT; post-anneal ~ 300 °C	Glass/Al₂O₃/sapphire/PI; ~1 μm	Hot growth raises $\sigma$ but trends Te-deficient; post-anneal improves texture and $PF$	$PF$ up to ~ 27.3 µW cm⁻¹ K⁻² (glass, post-anneal)	Best $PF$ via post-anneal; warns of Te loss during hot growth
Jitthamapirom (2019)^23^	DC vs. RF, Bi₂Te₃; pre-heat 150–350 °C	Polyimide; ~0.5 μm	Higher pre-heat improves (00ℓ) texture (DC); %Te decreases at highest pre-heat	$PF$ ~35 μW cm⁻¹ K⁻² (DC, 350 °C)	Flexible films; thermal window constrained by Te volatility
Norimasa (2021)^24^	DC, Bi₂Te₃; RT deposition; anneal to ~ 300 °C with 4–16 K min⁻¹ ramps	Al₂O₃; ~1 μm	Faster ramps preserve Te and enhance texture without excessive coarsening	$PF$ ~17.5 µW cm⁻¹ K⁻² (fast ramp); $\kappa$ ~1.08 W m⁻¹ K⁻¹; $ZT$ ~0.48	Post-anneal kinetics are a strong lever alongside ${T}_{sub}$
He (2020)^25^	DC, n-Bi₂Te₂.₆Se₀.₄; ~ 300 °C in-situ; in-situ anneal 10–70 min	Rigid substrate; ~0.8 μm	Anneal tunes carrier density; grain growth increases	$\sigma$ ~760–1030 S cm⁻¹; $S$ ↑; $PF$ ~4.5–4.9 µW cm⁻¹ K⁻² (RT)	Controlled thermal budget during/after growth maintains PF
Ao (2022)^26^	RF, Bi₂Te₃; ~300 °C; post electric-current pulses	Polyimide; ~0.58 μm	Current pulses densify microstructure and tune carriers	$\sigma$ up to ~ 2065 S cm⁻¹; $S$ ~−70 to − 80 µV K⁻¹ (est.); $PF$ ~10.7 µW cm⁻¹ K⁻²	Joule treatment is an effective alternative “thermal” lever
Zhou (2024)^27^	Magnetron, Bi₂Te₃ thick films; static 200 vs. 300 °C (1–5 h)	AlN; >10 μm	Clear $\sigma$ trends with growth temperature and time	$PF$ avg ~ 12 mW m⁻¹ K⁻² (≈ 120 µW cm⁻¹ K⁻², 313–453 K)	Thick-film focus; principles generalizable to thin films

## Experimental details

Bi_2_Te_3_ thin films were synthesized using DC magnetron sputtering, a widely utilized technique for achieving highly uniform and controlled thin-film depositions. A high-purity Bi_2_Te_3_ target (99.999%), supplied by Nanografi, with a 2-inch diameter, was used as the sputtering source. The deposition process was conducted at a DC power of 50 W in an argon atmosphere, ensuring a stable and controlled growth environment. To achieve high-quality film adhesion and minimize contamination, glass substrates underwent a rigorous multi-step cleaning process. First, they were washed with soap and deionized water to remove surface debris. This was followed by sequential ultrasonic treatment in ethanol, acetone, and distilled water, each for 20 min, to eliminate organic residues. Finally, the cleaned substrates were air-dried using an air pump.

Following the cleaning process, substrates were securely positioned inside the sputtering chamber. The chamber conditions were precisely controlled, with a base pressure of $8.5\times{10}^{-5}$ Torr to ensure a contamination-free environment and a working pressure of $6\times{10}^{-3}$ Torr, maintained with a high-purity argon gas flow to sustain plasma stability. The deposition time was set to 300 s to achieve a film thickness of 450 nm, which was monitored and controlled using a quartz crystal sensor integrated into the sputtering system. To systematically examine the influence of substrate temperature on the structural, optical, and thermoelectric properties of Bi_2_Te_3_ thin films, depositions were conducted at four different substrate temperatures: Room Temperature (RT), 100 °C, 200 °C, and 300 °C. These variations allowed for an in-depth understanding of thermal effects on film crystallinity, morphology, and electronic transport behavior.

A comprehensive suite of advanced characterization techniques was employed to evaluate the morphological, structural, and optical properties of the Bi_2_Te_3_ thin films. The surface morphology and topography were analyzed using Field Emission Scanning Electron Microscopy (FESEM) (Zeiss Sigma 300-HV) to examine the surface texture, grain distribution, and uniformity of the films. The structural characterization of the films was carried out using X-ray Diffraction (XRD; Bruker AXS) with CuKα radiation ($\lambda$ = 1.5418 Å) to determine the crystallinity, phase composition, and preferred orientation of the deposited films. The optical absorption and transmission spectra of the films were measured using a Shimadzu UV-1800 UV-Vis spectrophotometer in the 300–1100 nm wavelength range to evaluate their bandgap energy and optical performance.

This systematic experimental approach ensures a thorough investigation into the role of substrate temperature in tailoring the microstructure and thermoelectric performance of Bi_2_Te_3_ thin films, laying the foundation for their potential applications in next-generation energy harvesting devices.

## Results and discussions

### XRD analysis

The XRD patterns (Fig. 1) confirm the formation of crystalline Bi₂Te₃ thin films deposited at different substrate temperatures. For RT and 100 °C, the diffraction peaks match well with the Bi₂Te₃ reference pattern (Ref. code: 00-015-0863), indicating successful phase formation. For 200 °C and 300 °C, no distinct peaks attributable to elemental Bi or Te are observed within the detection limit of the present XRD measurements. However, the systematic shifts of the dominant Bi₂Te₃ peak together with the compositional trend from EDS are consistent with increasing Te deficiency and deviation from ideal stoichiometry at elevated substrate temperature; any minor secondary phases, if present, are below the XRD detection limit.

As shown in Fig. 1, the main peak shifts to a lower 2θ from RT to 200 °C and then shifts slightly back to a higher 2θ at 300 °C. The corresponding change in interplanar spacing was evaluated using Bragg’s law:

1 $$n\lambda=2dSin\left(\theta\right)$$

Where $\theta=\left(2{\uptheta}\right)/2$ is the peak position, $n=1$, λ = 1.5406 Å, and $d$ represents the interplanar spacing in the crystal structure. The calculated *d*-spacing (Table 2) increases from 0.316 nm (RT) to 0.322 nm (200 °C), consistent with the shift to lower 2θ, and then decreases slightly to 0.321 nm at 300 °C, consistent with the shift to higher 2θ. The small reduction in *d*-spacing at 300 °C is consistent with Te volatility at higher substrate temperature and the associated change in lattice spacing^28^.

The crystallite size was estimated using the Debye-Scherrer equation:

2 $$D=\frac{k\lambda}{\beta\mathrm{c}\mathrm{o}\mathrm{s}\left({\uptheta}\right)}$$

Where $\beta$ represents the full width at half maximum (FWHM), $\lambda$=1.5406 Å is the X-ray wavelength, $k$ is the Scherrer constant (typically $k=0.9$). Lattice strain ($\epsilon$) is calculated using the following equation^38^:

3 $$\epsilon=\frac{\beta\mathrm{c}\mathrm{o}\mathrm{s}\left({\uptheta}\right)}{4}$$

Where $\epsilon$ represents the effective strain.

Additionally, the dislocation density ($\delta$), defined as the length of dislocation lines per unit volume of the crystal, can be expressed as follows^29^:

4 $$\delta=\frac{1}{{D}^{2}}$$

Table 2 shows that the crystallite size increases with substrate temperature up to 200 °C (maximum *D*), accompanied by reduced lattice and dislocation density, indicating improved crystalline quality. At 300 °C, the crystallite size decreases, and the lattice increases relative to 200 °C, suggesting a deterioration in crystalline quality at the highest substrate temperature. The enhanced intensity/narrowing of the preferred Bi₂Te₃ peak near 27–28° further supports the conclusion that the highest crystallinity among the investigated conditions occurs at 200 °C. A similar “optimal temperature window” has been reported for related chalcogenide films, where peak intensity and FWHM depend strongly on substrate temperature^30^.

**Table 2:** XRD-derived structural parameters of Bi₂Te₃ thin films deposited at various substrate temperatures.

Sample	Peak position (2θ, °)	FWHM (°)	Crystallite size, D_XRD_ (nm)	Lattice strain, ε (%)	Dislocation density, δ (*10^14^ m^− 2^)	$d-$spacing (nm)
RT	28.164	0.88	9.31	1.53	115.4	0.316
100	27.852	0.687	11.91	1.21	70.45	0.320
200	27.627	0.222	36.85	0.39	7.36	0.322
300	27.735	0.290	28.22	0.51	12.56	0.321

**Fig. 2:**
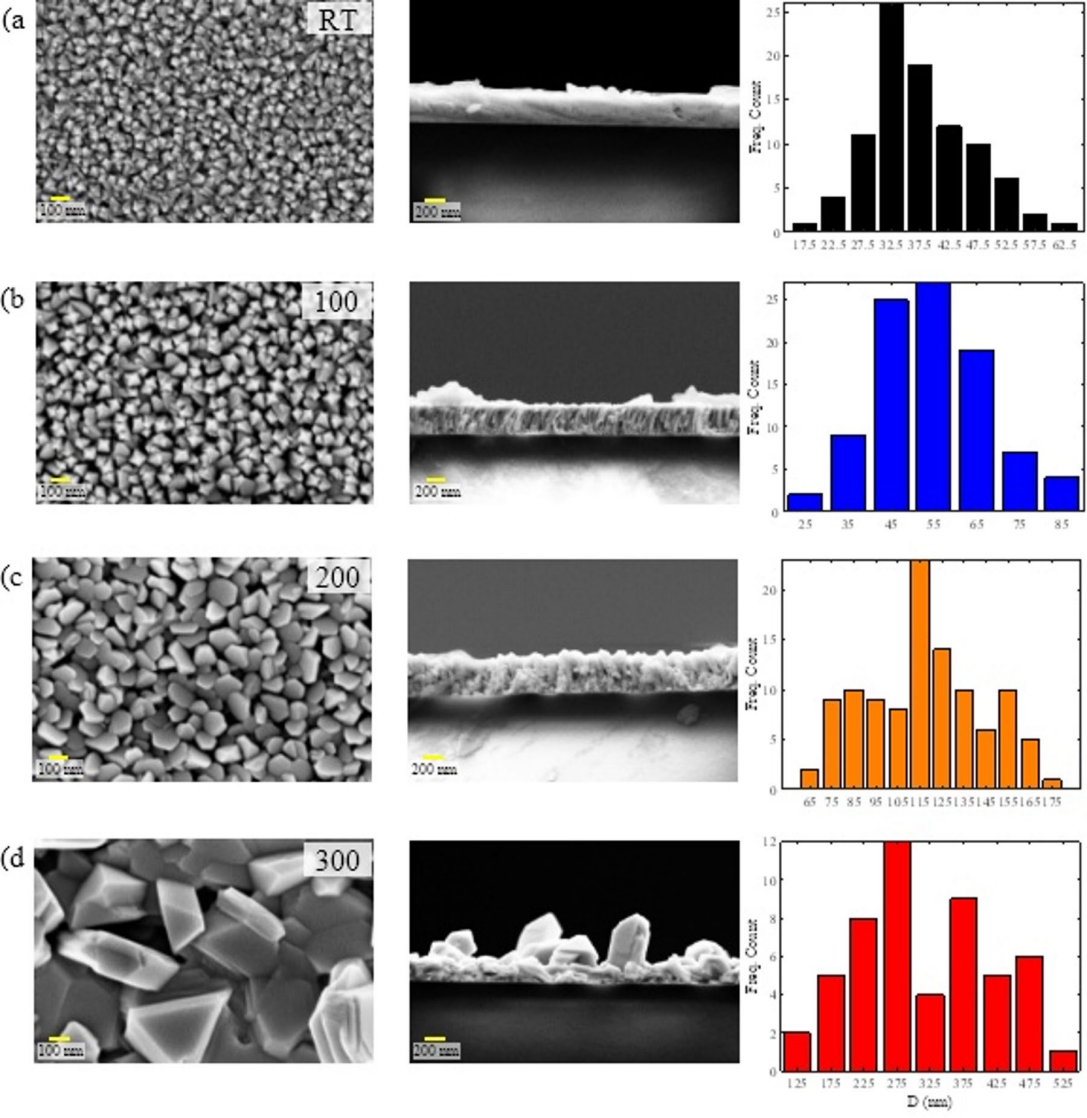
Top-view (left) and cross-sectional (middle) FESEM images, along with grain-size distribution histograms (right), of Bi₂Te₃ thin films deposited at RT (**a**), 100 °C (**b**), 200 °C (**c**), and 300 °C (**d**). The scale bar for the top-view images is 100 nm, and for the cross-sectional images is 200 nm, which are shown in yellow bars.

### FESEM analysis

Figure 2 presents top-view (left) and cross-sectional (middle) FESEM images of Bi₂Te₃ thin films deposited at RT, 100 °C, 200 °C, and 300 °C, together with the corresponding grain-size distribution histograms (right). Clear morphology evolution is observed with increasing substrate temperature. At RT, the film consists of densely packed fine grains that form a compact, continuous surface. Increasing the substrate temperature to 100 °C produces moderate grain coarsening while maintaining good surface coverage. At 200 °C, grains become more faceted and well-defined, consistent with improved crystalline ordering; however, local roughness becomes more pronounced. At 300 °C, grain coarsening is substantial, and the microstructure becomes increasingly heterogeneous, with more pronounced voided/porous features and reduced compactness. Such morphological changes can influence thermoelectric transport through competing mechanisms: larger grains may reduce grain-boundary scattering (potentially benefiting σ), whereas increased heterogeneity/porosity can reduce electrical connectivity and introduce additional carrier scattering.

Cross-sectional FESEM images indicate that the film thickness remains approximately ~ 450 nm for all samples, suggesting that the deposition rate is broadly similar over the investigated substrate temperature range.

To quantify microstructural evolution, grain size was measured from the top-view FESEM images using Digimizer software. The grain-size distributions were analyzed using a log-normal function^31^, and the extracted mean grain size ⟨D*g*⟩ and standard deviation σ are summarized in Table 3. The mean grain size increases from 35.63 ± 0.81 nm (RT) to 53.97 ± 0.66 nm (100 °C) and 117.44 ± 2.15 nm (200 °C), reflecting enhanced adatom mobility and grain growth with increasing substrate temperature. At 300 °C, the mean grain size increases markedly to 340.28 ± 176.21 nm, accompanied by a very large dispersion, indicating strong coarsening and pronounced non-uniformity due to grain agglomeration and the development of large faceted grains. Overall, 200 °C provides a favorable compromise where grain growth and crystalline ordering improve substantially while the microstructure remains comparatively more uniform than at 300 °C, consistent with the transport trends discussed below. (Fig. 2, right panels).

**Table 3:** Grain-size statistics extracted from top-view FESEM images (Digimizer) using log-normal analysis^31^.

SamplE	Mean grain size, <Dg> ± σ (nm)
RT	35.63 ± 0.81
100	53.97 ± 0.66
200	117.44 ± 2.15
300	340.28 ± 176.21

**Fig. 3:**
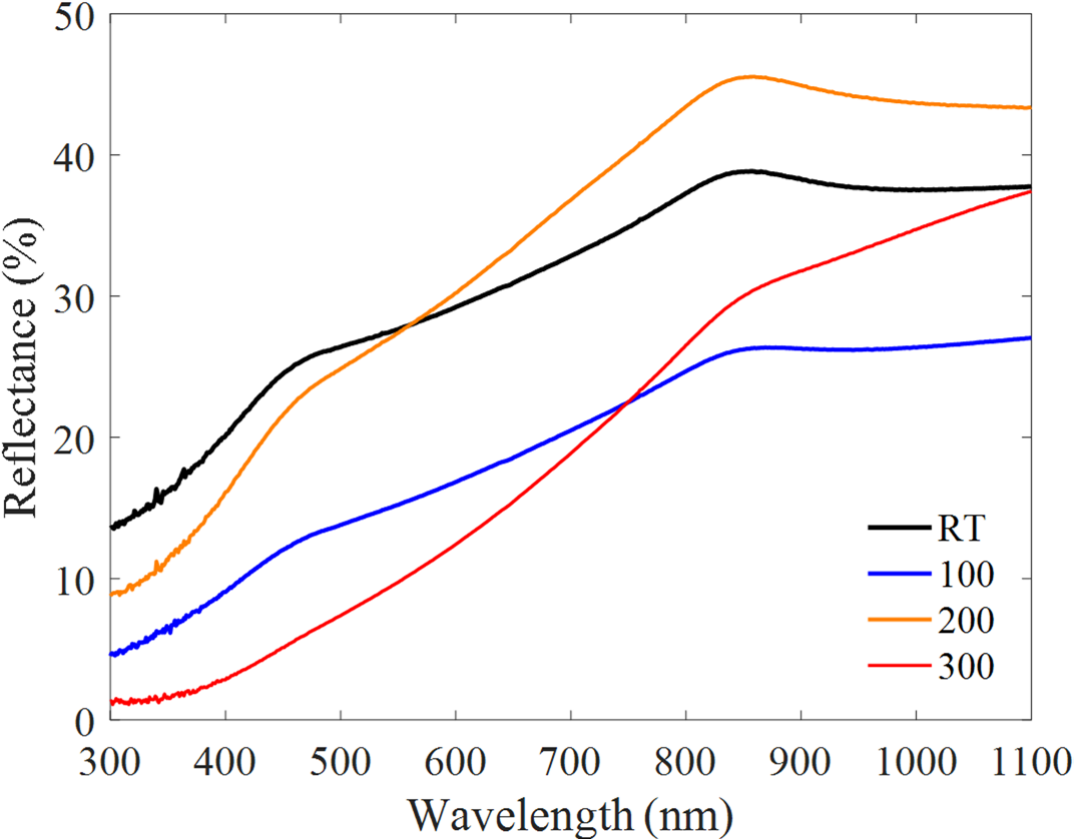
Reflectance spectra of Bi₂Te₃ thin films deposited at RT, 100 °C, 200 °C, and 300 °C measured in the 300–1100 nm wavelength range.

### EDS analysis

EDS results show a clear dependence of film stoichiometry on substrate temperature (Table 4). Films deposited at RT and 100 °C exhibit Te contents close to the nominal Bi₂Te₃ composition, indicating minimal deviation from stoichiometry. With increasing substrate temperature, Te content decreases progressively (from 56.63 at% at RT to 52.85 at% at 300 °C), with a corresponding increase in Bi fraction, indicating that elevated substrate temperature promotes Te loss and drives the films toward a more Bi-rich composition. This trend is consistent with the relatively high volatility of Te during high-temperature sputtering and is also consistent with the lattice-spacing trend and transport behavior at the highest deposition temperature.

Combining FESEM and EDS, the thermal budget is seen to influence both microstructure and composition: moderate heating (≈ 200 °C) promotes grain growth and improved ordering while maintaining a comparatively smaller deviation from stoichiometry than at 300 °C, whereas the highest temperature produces pronounced grain coarsening/non-uniformity together with stronger Te loss. Similar temperature-dependent behavior has been reported for related telluride thin films; for example, Lin et al.^30^ observed Te separation at lower substrate temperatures during Sb₂Te₃ growth, followed by improved compound formation at an intermediate optimum temperature^30^.

**Table 4:** EDS elemental composition of Bi2Te3 thin films deposited at different substrate temperatures.

Sample/element	Te (at%)	Bi (at%)
RT	56.63	43.37
100	56.52	43.48
200	55.56	44.44
300	52.85	47.15

### Optical properties

To investigate the effect of substrate temperature on the optical response, the reflectance spectra of the films were measured in the 300–1100 nm wavelength range (Fig. 3). For all samples, the reflectance increases with wavelength. Clear differences are observed among deposition temperatures: the film deposited at 200 °C exhibits the highest reflectance over most of the measured range, whereas the 300 °C film shows the lowest reflectance. This trend is consistent with the microstructural evolution observed by FESEM, where the 300 °C film becomes more heterogeneous and less compact, which can enhance light trapping and scattering and thereby reduce the measured reflectance compared with more compact films.

**Fig. 4:**
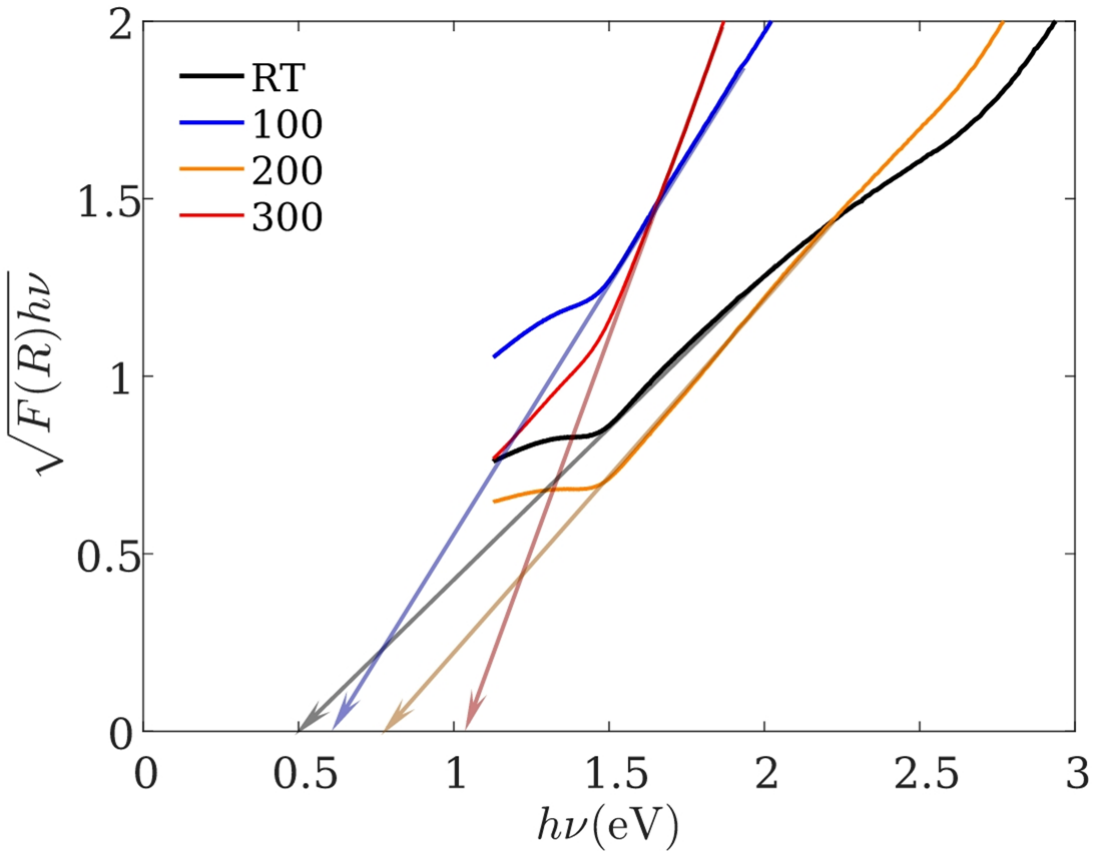
Tauc-type plot based on the Kubelka–Munk transform, ${\left[F\left(R\right)hv\right]}^{\frac{1}{2}}$ versus $hv$, used to estimate the apparent optical bandgap ${E}_{g,app}$ in the 300–1100 nm window for Bi₂Te₃ thin films deposited at different substrate temperatures^32^.

The apparent optical band gap ${E}_{g,\mathrm{a}\mathrm{p}\mathrm{p}}$, was estimated from the reflectance data using the Kubelka–Munk function and Tauc-type analysis^32^. The Kubelka–Munk transform is given by:

5 $$F\left(R\right)=\frac{{\left(1-R\right)}^{2}}{2R}$$

where $R$ represents reflection. The apparent bandgap was then obtained by plotting ${\left[F\left(R\right)hv\right]}^{\frac{1}{2}}$ versus $hv$ and extrapolating the linear region to the energy axis (Fig. 4). The extracted ${E}_{g,\mathrm{a}\mathrm{p}\mathrm{p}}$ Values are summarized in Table 5.

**Table 5:** Apparent optical bandgap ${E}_{g,app}$ (eV) of Bi₂Te₃ thin films extracted from Kubelka–Munk/Tauc-type analysis of reflectance data in the 300–1100 nm window^32^.

Sample	Bandgap (eV)
RT	0.48
100	0.56
200	0.78
300	1.85

Importantly, Bi₂Te₃ is a narrow-gap semiconductor (intrinsic electronic bandgap ~ 0.15 eV, i.e., in the mid-infrared), and UV–Vis measurements in the 300–1100 nm range do not probe the fundamental band edge. Therefore, the values reported here represent an apparent optical bandgap within the measured spectral window, which is sensitive to composition, disorder, and carrier-related optical response, and should not be interpreted as intrinsic bandgap widening.

Within this interpretation, ${E}_{g,\mathrm{a}\mathrm{p}\mathrm{p}}$ increases with substrate temperature from 0.48 eV (RT) to 1.85 eV (300 °C) (Table 5). The observed blue shift of ${E}_{g,\mathrm{a}\mathrm{p}\mathrm{p}}$ with increasing substrate temperature can be rationalized by several concurrent effects. First, EDS indicates progressive Te deficiency at elevated substrate temperatures (Table 4), and variations in the Te/Bi ratio are known to influence the optical response and electronic structure of Bi₂Te₃-based films^33,34^. Second, disorder and defect-related absorption-tail states associated with non-stoichiometric growth can modify the absorption onset and the energy extracted from Tauc-type plots. Third, substrate-temperature-driven changes in carrier concentration may lead to band-filling (Burstein–Moss-type) effects in degenerate or highly doped films, producing an apparent widening of the optical gap. Additionally, microstructural roughness and porosity can alter light scattering and effective optical pathlength, which may influence the extracted apparent bandgap. Accordingly, the optical bandgap values reported here should be interpreted as comparative metrics describing the evolution of the apparent absorption edge within the measured UV–Vis window, consistent with the structural and compositional trends discussed above.

**Fig. 5:**
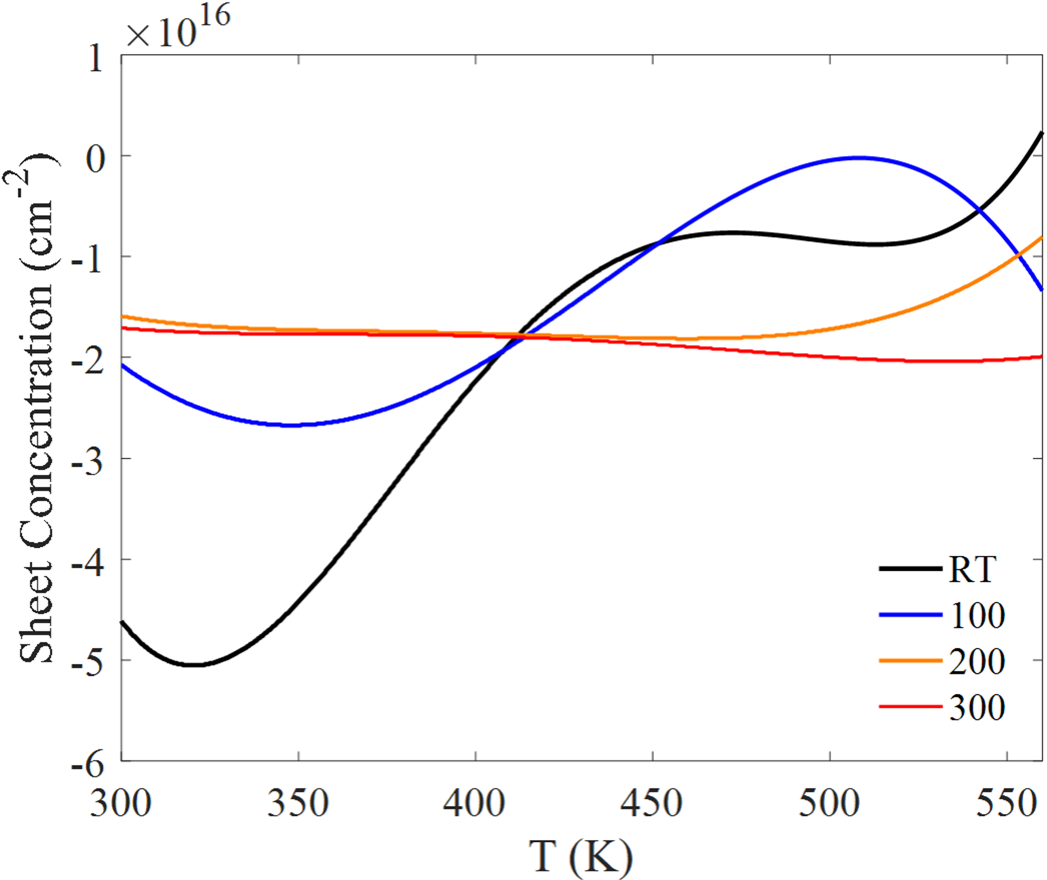
The sheet concentration of the samples as a function of temperature.

### Electrical properties

**Fig. 6:**
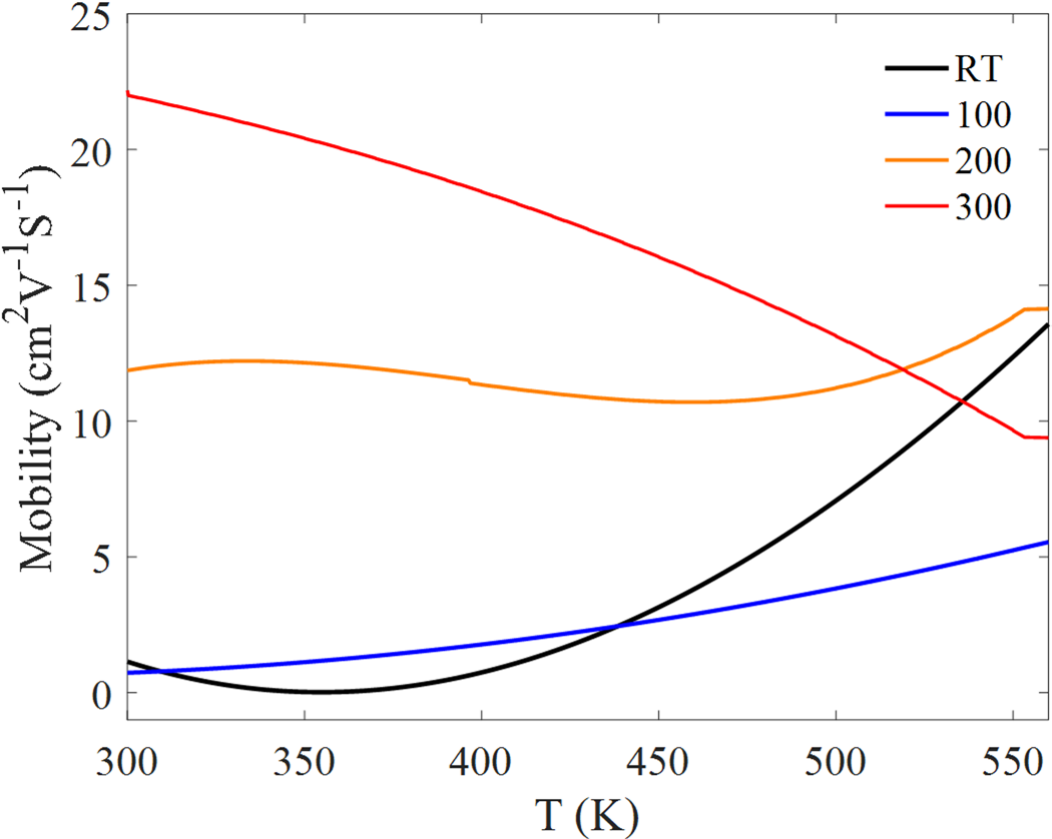
The mobility of the samples as a function of temperature.

**Fig. 7:**
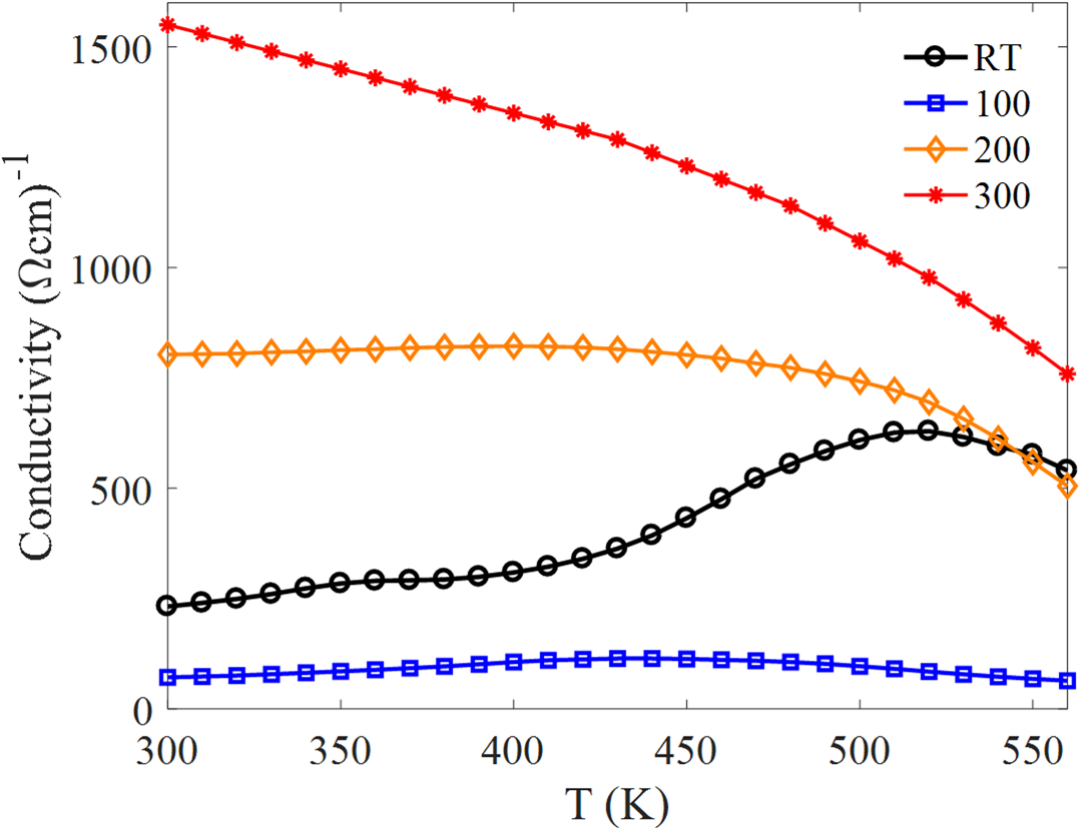
The electrical conductivity of the samples as a function of temperature.

The electrical transport behavior of the Bi₂Te₃ thin films was evaluated as a function of temperature using Hall effect measurements in a Van der Pauw configuration^35^. Films deposited at different substrate temperatures exhibit distinctly different transport trends, reflecting the combined influence of microstructure (grain size/connectivity) and composition (Te-loss-driven deviation from stoichiometry). Key transport parameters, including sheet carrier concentration (Fig. 5), carrier mobility (Fig. 6), and electrical conductivity (Fig. 7), show a strong dependence on substrate temperature.

In Fig. 5, the sheet carrier concentration is presented as a signed quantity, where the sign reflects the sign of the Hall coefficient: negative values correspond to electron-dominated (n-type) transport and positive values correspond to hole-dominated (p-type) transport. Over most of the measurement range, the films exhibit predominantly negative values, indicating electron-dominated conduction under the adopted sign convention. The temperature evolution of $\left|{n}_{s}\right|$ is most pronounced for the RT and 100 °C films, whereas the 200 °C and 300 °C films show comparatively steadier behavior.

Carrier mobility (Fig. 6) also depends strongly on deposition temperature. The RT and 100 °C films show lower mobility at low temperature, consistent with stronger grain-boundary scattering expected for finer-grained microstructures, while the 200 °C and 300 °C films exhibit higher mobility, consistent with improved grain connectivity and reduced grain-boundary scattering (Table 3). With increasing measurement temperature, mobility trends reflect the competition between phonon scattering and thermally assisted carrier transport. Notably, the RT film shows a strong increase in mobility with temperature, which can be associated with thermally assisted carrier delocalization, whereas the 300 °C film shows a decline in mobility at higher temperature, consistent with increased phonon scattering and/or defect-related scattering at elevated thermal loads.

Electrical conductivity (Fig. 7) follows the combined evolution of carrier concentration and mobility. In general, conductivity decreases with increasing temperature for the 100 °C, 200 °C, and 300 °C films, consistent with enhanced phonon scattering at elevated temperature. The RT film exhibits an increase in conductivity up to ~ 520 K, which coincides with its strong mobility increase and compensates for changes in carrier concentration. A similar temperature-dependent decline in electrical conductivity has been reported for Bi₂Te₂.₇Se₀.₃ bulk materials, highlighting the critical influence of carrier scattering and microstructure on thermoelectric transport^36^.

Overall, films deposited at higher substrate temperatures (200 °C and 300 °C) exhibit superior conductivity, attributable to improved crystalline ordering, larger grain size, and more efficient charge-transport pathways. In contrast, films deposited at RT and 100 °C show reduced conductivity due to finer grains and increased grain-boundary scattering. The conductivity values obtained in this work are consistent with, and in some cases exceed, those reported in earlier studies^37,38^, reaffirming the critical role of substrate temperature in tuning electrical transport in sputtered Bi₂Te₃ thin films^39^.

### Seebeck coefficient and thermoelectric properties

**Fig. 8:**
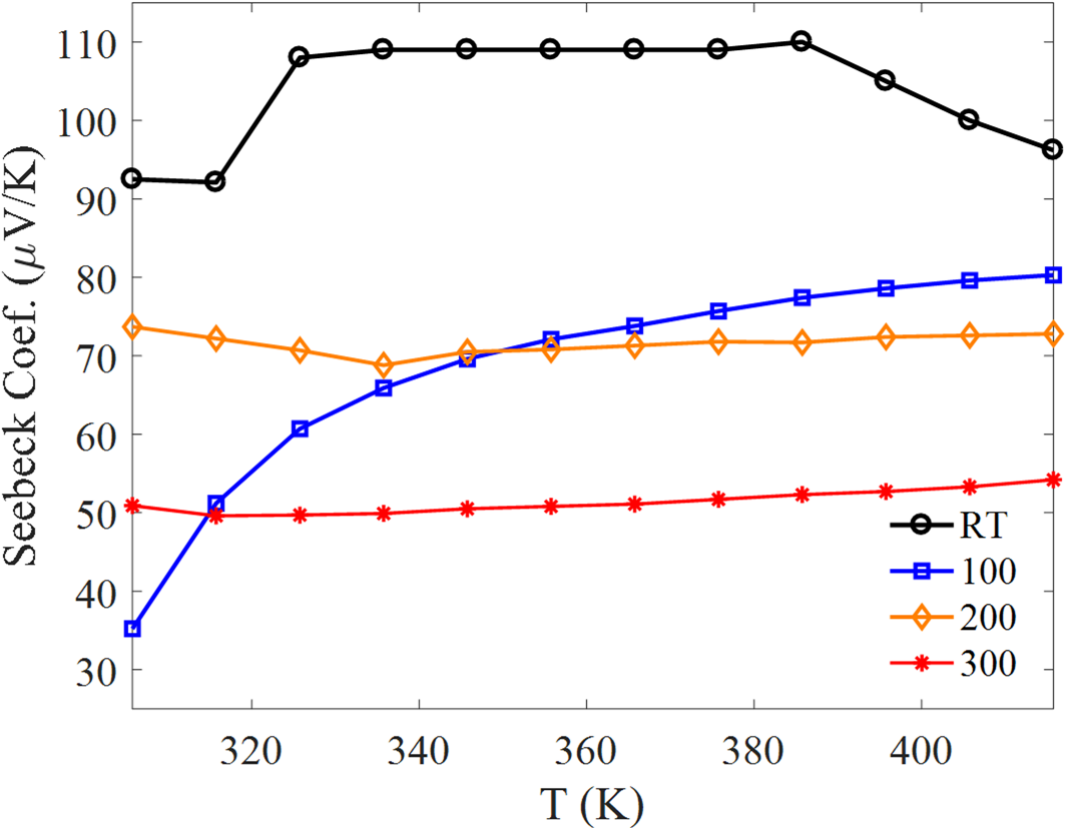
Seebeck coefficient of Bi₂Te₃ thin films deposited at different substrate temperatures as a function of temperature.

The Seebeck coefficient shows the expected trade-off with electrical conductivity, consistent with typical thermoelectric behavior: samples with higher conductivity generally exhibit a smaller Seebeck magnitude. The Seebeck coefficient (Fig. 8) is a key parameter for thermoelectric assessment because it directly enters the power factor ($PF = S^{2} \sigma$) and the figure of merit (ZT)^40,41^. In the present study, Bi₂Te₃ thin films deposited at RT, 100 °C, 200 °C, and 300 °C exhibit a strong dependence of Seebeck response on composition (Te loss), defect chemistry, and transport parameters.

Carrier type and sign consistency are essential when interpreting Hall and Seebeck data. Under the standard convention, n-type transport corresponds to a negative Hall coefficient and a negative Seebeck coefficient, while p-type transport corresponds to positive values for both. Since the Hall results (Fig. 5) are predominantly negative for most samples and temperatures, the Seebeck coefficient should be reported using a sign convention consistent with electron-dominated transport. If the plotted Seebeck values were exported or presented as magnitudes, the sign should be adjusted accordingly in the revised figure/caption so that Hall and Seebeck consistently indicate the same carrier type.

The temperature and deposition-temperature dependence of Seebeck can be rationalized within the degenerate-semiconductor framework. The Seebeck coefficient is related to the energy dependence of conductivity near the Fermi level (Mott relation)^45,46^:

6 $$S = \left. { - \frac{{\pi ^{2} k_{B}^{2} T}}{{3\left| e \right|}}\frac{{\partial \ln \sigma \left( E \right)}}{{\left( {\partial E} \right)x}}} \right|_{{E_{F} }} = - \frac{{\pi ^{2} k_{B}^{2} m^{*} T}}{{\left( {3\pi ^{2} } \right)^{{2/3}} \left| e \right|\hbar ^{2} n^{{2/3}} }}$$

where $e$ is the electronic charge, $n$ carrier density, ${k}_{B}$ the Boltzmann constant, $\hslash$ the reduced Planck constant, $\sigma \left(E\right)$ the energy-dependent conductivity, ${E}_{F}$ the Fermi energy, $T$ temperature, and ${m}^{*}$ the effective mass of charge carriers^46^.

The magnitude of $\left|S\right|$ commonly decreases with increasing carrier concentration (Pisarenko-type behavior). In this work, the RT-deposited film exhibits the highest Seebeck magnitude, whereas the 300 °C film shows the lowest magnitude (Fig. 8). This trend is consistent with increasing Te-loss-driven deviation from stoichiometry at elevated substrate temperature (Table 4), which is expected to modify carrier density and scattering. Defects such as Te vacancies and Bi/Te antisites are known to strongly influence carrier concentration and carrier type in Bi₂Te₃ films^42^, and defect interactions under non-stoichiometric growth can modulate transport behavior^39,43,44^. Importantly, while XRD does not show detectable elemental Bi or Te peaks within measurement limits, peak shifts/lattice-spacing trends together with EDS support increasing deviation from ideal stoichiometry at higher substrate temperature.

**Fig. 9:**
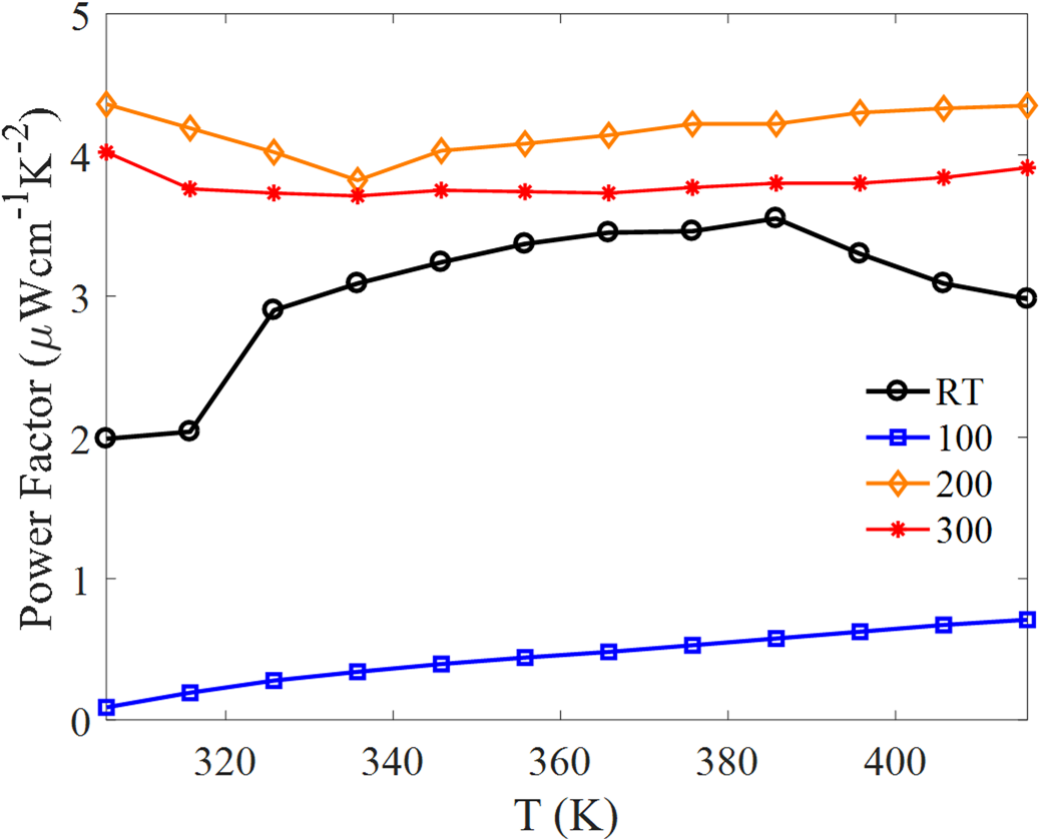
Power factor of Bi2Te3 thin films deposited at different substrate temperatures as a function of temperature.

The power factor, $PF = S^{2} \sigma$^48^, is plotted in Fig. 9. $PF$ exhibits a clear dependence on substrate temperature, with the maximum value of ~ 4 µW cm⁻¹ K⁻² obtained for films deposited at 200 °C. This enhancement reflects an optimal balance between conductivity and Seebeck magnitude, consistent with improved grain connectivity (Table 3) and only moderate stoichiometry deviation compared with the 300 °C film (Table 4). RT-deposited films exhibit relatively high Seebeck magnitude but lower $PF$ due to reduced conductivity associated with limited grain growth and enhanced grain-boundary scattering. Films deposited at 100 °C show the lowest $PF$ due to the unfavorable combination of low conductivity and modest Seebeck response. At 300 °C, $PF$ decreases slightly compared to 200 °C because the higher conductivity is offset by a substantially reduced Seebeck magnitude, consistent with stronger Te loss/Bi-rich deviation and increased microstructural heterogeneity at the highest substrate temperature.

The obtained *PF* values are reasonable for sputtered Bi₂Te₃ thin films; however, direct comparison with bulk Bi₂Te₃ is avoided because bulk and thin-film systems can differ substantially in microstructure and charge-transport mechanisms. The identified intermediate-temperature optimum is consistent with prior reports on sputtered Bi₂Te₃ films^25^. Similar *PF* stability over temperature has also been reported for Bi₂Te₃ thin films prepared by other deposition routes^49, 50^. Thermal conductivity (κ) was not measured in this study; therefore, the discussion is limited to *PF* trends and does not attempt to calculate the figure of merit ZT. Future work will incorporate direct κ measurements (e.g., 3ω or laser-flash methods) to enable full ZT evaluation.

## Conclusion

This work systematically evaluated how substrate temperature (RT–300 °C) governs the growth–structure–composition–transport relationships in ~ 450 nm Bi₂Te₃ thin films deposited on glass by single-target DC sputtering. FESEM shows a clear microstructural evolution with increasing substrate temperature: grains coarsen and become more faceted from RT to 200 °C, while deposition at 300 °C produces pronounced grain coarsening with large dispersion and a more heterogeneous, less compact morphology. Cross-sectional imaging indicates that the film thickness remains approximately ~ 450 nm across all samples, confirming that the observed property changes arise primarily from microstructural and compositional differences rather than thickness variations.

XRD confirms the formation of crystalline Bi₂Te₃ for all deposition temperatures, with systematic peak shifts and lattice-spacing trends as substrate temperature increases. No distinct diffraction peaks corresponding to elemental Bi or Te were detected within the measurement limits; however, the combined XRD peak shifts and EDS results indicate increasing deviation from ideal stoichiometry at elevated substrate temperatures. EDS reveals a progressive decrease in Te content with increasing substrate temperature, consistent with Te volatility and a trend toward Bi-rich/Te-deficient composition at 300 °C.

Electrical transport reflects the interplay between carrier concentration and mobility. Films deposited at higher substrate temperatures exhibit higher conductivity, consistent with improved grain connectivity and reduced grain-boundary scattering, while the Seebeck response shows the expected trade-off with conductivity. The power factor, $PF={S}^{2}\sigma$, reaches its maximum value of ~ 4 µW cm⁻¹ K⁻² for films deposited at 200 °C, indicating that this condition provides the most favorable balance between electrical conductivity and Seebeck coefficient within the explored temperature window. In contrast, RT and 100 °C films exhibit reduced PF due to limited conductivity, and the 300 °C films show slightly lower *PF* than the 200 °C films because the gain in conductivity is offset by reduced Seebeck response, consistent with stronger Te loss and increased microstructural heterogeneity.

Optical measurements in the 300–1100 nm range reveal deposition-temperature-dependent changes in reflectance and an increasing apparent optical bandgap ${E}_{g,\mathrm{a}\mathrm{p}\mathrm{p}}$ extracted from Kubelka–Munk/Tauc-type analysis. These values represent an apparent absorption-edge metric within the measured window and should not be interpreted as the intrinsic Bi₂Te₃ electronic bandgap (mid-IR); nevertheless, the trend is consistent with the structural/compositional evolution observed by XRD and EDS.

Overall, the results demonstrate that substrate temperature is a primary processing lever for tailoring microstructure, stoichiometry, and thermoelectric transport in DC-sputtered Bi₂Te₃ thin films on glass. A substrate temperature of ~ 200 °C emerges as the most favorable condition in this study, delivering the highest *PF* through improved crystallinity and grain connectivity while limiting stoichiometry drift compared with 300 °C. Future work will focus on direct thermal conductivity measurements to enable ZT evaluation, improved control of Te loss (e.g., process optimization or compositional compensation), and stability testing under thermal cycling, alongside exploration of alternative substrates and integration-oriented device architectures.

## Data Availability

Data will be made available on reasonable request from the corresponding author, Dr Mahmood Akbari (email: mahmoa@unisa.ac.za).
